# Long Non-Coding RNA BANCR Promotes Proliferation in Malignant Melanoma by Regulating MAPK Pathway Activation

**DOI:** 10.1371/journal.pone.0100893

**Published:** 2014-06-26

**Authors:** Ruiya Li, Lingli Zhang, Lizhou Jia, Yan Duan, Yan Li, Lidao Bao, Na Sha

**Affiliations:** 1 Department of Dermatology, Inner Mongolia People's Hospital, Hohhot, Inner Mongolia Autonomous Region, China; 2 Department of Pathology, Inner Mongolia People's Hospital, Hohhot, Inner Mongolia Autonomous Region, China; 3 Department of Pathology, The Affiliated People's Hospital of Inner Mongolia Medical University, Hohhot, Inner Mongolia Autonomous Region, China; 4 Department of Dermatology, The Affiliated Hospital of Inner Mongolia Medical University, Hohhot, Inner Mongolia Autonomous Region, China; 5 Department of Pharmacy, The Affiliated Hospital of Inner Mongolia Medical University, Hohhot, Inner Mongolia Autonomous Region, China; University of Connecticut Health Center, United States of America

## Abstract

Long non-coding RNAs (lncRNAs) have been shown to be implicated in the complex network of cancer including malignant melanoma and play important roles in tumorigenesis and progression. However, their functions and downstream mechanisms are largely unknown. This study aimed to investigate whether BRAF-activated non-coding RNA (BANCR), a novel and potential regulator of melanoma cell, participates in the proliferation of malignant melanoma and elucidate the underlying mechanism in this process. We found that BANCR was abnormally overexpressed in human malignant melanoma cell lines and tissues, and increased with tumor stages by quantitative PCR. BANCR knockdown induced by shRNA transfection significantly inhibited proliferation of tumor cells and inactivated MAPK pathway, especially by silencing the ERK1/2 and JNK component. Moreover, combination treatment of BANCR knockdown and suppression ERK1/2 or JNK (induced by specific inhibitors U0126 or SP600125 respectively) produced synergistic inhibitory effects *in vitro*. And the inhibitory effects induced by ERK1/2 or JNK could be rescued by BANCR overexpression. By tumorigenicity assay in BALB/c nude mice, we further found that BANCR knockdown inhibited tumor growth *in vivo*. In addition, patients with high expression of BANCR had a lower survival rate. Taken together, we confirmed the abnormal upregulation of a novel lncRNA, BANCR, in human malignant melanoma. BANCR was involved in melanoma cell proliferation both *in vitro* and *in vivo*. The linkage between BANCR and MAPK pathway may provide a novel interpretation for the mechanism of proliferation regulation in malignant melanoma.

## Introduction

Melanoma is the most aggressive type of skin cancer, characterized by a rapid progression, metastasis to regional lymph nodes and distant organs as well as a limited efficiency of therapeutics [1]. Although it accounts for only about 4% of all dermatological cancers, it contributes to more than 80% death of skin cancer patients [2]. Therefore, it is necessary to understand thoroughly which factors involved in the development and progression of melanoma. In addition, though many molecules contributing to invasion and migration have been reported [3], [4], the precise mechanisms underlying proliferation, the first step of tumor pathogenesis, remain to be fully elucidated.

Recently, growing attention has been given to a class of non-protein-coding RNAs (ncRNAs), known as long non-coding RNAs (lncRNAs), which participates in cell fate determination and disease pathogenesis [5], [6]. LncRNAs are non-coding RNAs greater than 200 nucleotides in length and can regulate gene expression in different biological processes transcriptionally or post-transcriptionally. And its dysregulation has been observed under many pathological conditions including cancers, heart diseases and Alzheimer disease [7]. For example, HOTAIR seems to modulate tumor invasiveness by enhancing PRC2-mediated suppression of metastasis [8]. PRNCR1 and PCGEM1 can bind successively to androgen receptor and strongly enhance androgen receptor-mediated gene activation and proliferation in prostate cancer [9]. And dysregulation of HNF1A-AS1 participates in oesophageal tumorigenesis by modulation of chromatin and nucleosome assembly as well as by induction of cancer-related H19 [10].

In previous study, Flockhart RJ et al. [11] and McCarthy N [12] identified a previously unstudied but widely expressed lncRNA, BRAF-activated non-coding RNA (BANCR), as playing a potentially functional role in melanoma cell migration by RNA-sequencing. In the present study, we confirmed the role of BANCR in the proliferation of malignant melanoma, and aimed to elucidate the contribution of MAPK pathway in this process. Our data demonstrated that BANCR can promote melanoma proliferation via activating ERK1/2 and JNK MAPK pathway both *in vitro* and *in vivo*. The linkage of BANCR and the MAPK pathway may unravel a novel mechanism in the regulation of melanoma proliferation.

## Materials and Methods

### Tissue samples

Tissues were obtained from patients (62 male and 41 female) who underwent surgery at Department of Dermatology, Inner Mongolia People's Hospital from 2005 to 2010. The study was carried out in accordance with the institutional ethical guidelines and the use of human skin tissues was approved by the Medical Ethics Committee of Inner Mongolia People's Hospital (IMP study ID H05-561321). Every patient involved in the study was asked to sign a piece of written informed consent which has been approved by the ethics committee of Inner Mongolia People's Hospital, and all the consents were saved by the ethics committee. The study was conducted according to the principles expressed in the Declaration of Helsinki. Skin tissues from 12 patients with melanocytic nevus (matched by gender and age) were collected as controls. All samples were snap-frozen in liquid nitrogen, then stored at −80°C for further use. Patient descriptions are detailed in table 1. Clinical follow-up data were available for 72 patients, while 31 patients were excluded for lack of information.

**Table 1:** Association between BANCR and clinicopathological parameters of patients.

Characteristic	N (%)	BANCR	P value
All cases	103 (100)		
Age			0.092
≤60 y	48 (46.6)	4.8(3.4–7.6)	
>60 y	55 (53.4)	5.1(3.0–8.2)	
Histologic type			0.316
Superficial spreading	33 (32.0)	5.2(2.8–7.7)	
Nodular	25 (24.3)	4.9(2.5–6.5)	
Acral lentiginous	21 (20.4)	5.4(3.0–7.1)	
Lentigo maligna	7 (6.8)	4.6(3.1–6.9)	
Other	17(16.5)	5.0(2.2–8.3)	
Location			0.081
Head and neck	53 (51.5)	4.2(2.3–6.6)	
Trunk	27 (26.2)	3.9(2.5–7.3)	
Extremities	23 (22.3)	4.3(3.1–8.6)	
TNM classification			0.017
I–II	20 (19.4)	2.2(1.8–3.5)	
III	70 (68.0)	4.2(3.9–6.1)	
IV	13 (12.6)	6.8(6.3–8.3)	

### Cell culture and treatment

Five human melanoma cells lines, including A-375, 1205Lu, UACC903, CHL-1 and sk-mel-5, were purchased from American Type Culture Collection. Cells were maintained in RPMI-1640 medium (A-375, 1205Lu) or DMEM medium (UACC903, CHL-1 and sk-mel-5) respectively supplemented with 10% fetal bovine serum (Gibco, CA, USA) in a 37°C humidified atmosphere of 5% CO_2_.

Knockdown of BANCR was induced by transfection with BANCR shRNA (Genepharma, Shanghai, China) using Lipofectamine2000 (Invitrogen, CA, USA). The target sequences were as follows: shRNA #1: 5′-CGGAAATAGACTGCAGCAC CAA-3′; shRNA #2: 5′-CCTTTATGGATTCAACTGTAAT-3′. Ectopic expression of BANCR was achieved through pCDNA3.1-BANCR transfection using Lipofectamine2000. Oligonucleotides for amplification of BANCR were as follows: sense: 5′- CAGGAAGGGGTGAATGAAGA-3′; antisense: 5′- CCAGTGCAGGGTAATGTGTG-3′.

Stable transfectants were selected in medium containing 600 µg/mL G418 (Invitrogen, CA, USA) and used in further assays or RNA/protein extraction.

For treatment with pharmacological inhibitors, the overnight starved cells were kept in complete medium containing 10 µM U0126 (inhibitor of the upstream ERK1/2 activator MEK1/2, Sigma-Aldrich, MO, USA) or 20 µM SP600125 (JNK inhibitor, Sigma-Aldrich, MO, USA). DMSO was used as a control. Medium containing inhibitor was renewed every day.

### RNA extraction and quantitative real-time PCR analysis

Total RNA was extracted from tissues or cells using Trizol reagent (Invitrogen, CA, USA). BANCR expressions were measured by SYBR green qPCR assay (Takara, Dalian, China) in triplicate. Primer sequences were as follows: BANCR, forward: ACAGGACTCCATGGCAAACG, reverse: ATGAAGAAAGCCTGGTGCAGT; GAPDH, forward: ACCACAGTCCATGCCATCAC, reverse: TCCACCACCCTGTTGCTGTA. Data were processed using 2-^ΔΔ^CT method and normalized to GAPDH expression.

### Protein extraction and western blot analysis

Cells were lysed in RIPA buffer with protease inhibitors and phosphatase inhibitors. Protein was loaded onto a SDS-PAGE minigel and transferred onto PVDF membrane. The blots were probed with primary antibodies (Cell signal technology, MA, USA) followed by the HRP-conjugated secondary antibody. Signals were visualized using ECL Substrates (Millipore, MA, USA). GAPDH served as the loading control.

### CCK-8 cell proliferation assay

Cell proliferation rates were measured using Cell Counting Kit-8 (CCK-8, Beyotime, Hangzhou, China). 0.5×10^4^ cells were seeded in each 96-well plate for 24 h, treated with or without U0126 or SP600125, and further incubated for 24 h, 48 h and 72 h respectively. After 10 µl CCK-8 reagent was added to each well, the plate was returned to the incubator for 1 h, the absorbance at 450 nm was measured using an enzyme-linked immunosorbent assay plate reader.

### Tumorigenicity assay in nude mice

This study was carried out in strict accordance with the recommendations in the Guide for the Care and Use of Laboratory Animals of Inner Mongolia People's Hospital. The protocol was approved by the Committee on the Ethics of Animal Experiments of Inner Mongolia People's Hospital (IMP study ID H05-561321).

A total of 24 male BALB/c nude mice (18–20 g, 5 weeks old, Animal Center of the Chinese Academy of Science, Shanghai, China) were randomized into three groups of eight each and housed 4 per cage under specific-pathogen-free conditions (room temperature-controlled at 24±2°C with a relative humidity of 60±5%, on a 12 h light/dark cycles) in the Animal Care Facility Service (Inner Mongolia People's Hospital). Standard rodent chow and fresh distilled water were supplemented every two days. Mice were anesthetized with intraperitoneal injection of sodium pentobarbital (0.3%, 50–60 mg/Kg) to relieve pain and then injected subcutaneously with 4×10^6^ cells into the flanks. Physiological parameters, including activity, food/water intake, skin, hair, excrement and mental state were monitored every day. And tumor volume and weight were monitored once every 5 days. Tumor volume was calculated according to the formula: tumor volume  =  length × (width) ^2^/2. After five weeks, mice (0/8 in group NC; 5/8 in group shRNA #1; 6/8 in group shRNA #2) presented symptoms including extremely slow activity, lethargy, hair loss and obvious weight loss, thus all the mice were sacrificed by pentobarbital overdose (1%) and exsanguination according to American Veterinary Medical Association guidelines for euthanasia.

### Statistical analysis

All values were expressed as mean ± SD and processed using the SPSS 13.0 software. Statistical significance was noted at P<0.05. Three independent experiments were performed. The differences among the groups in proliferation assays, western blot and PCR assays were estimated by Student's t-test or one-way ANOVA. The survival curves were plotted according to the Kaplan-Meier method and compared using the Log-rank test.

## Results

### BANCR is frequently upregulated in malignant melanoma tissues and cell lines

**Figure 1:**
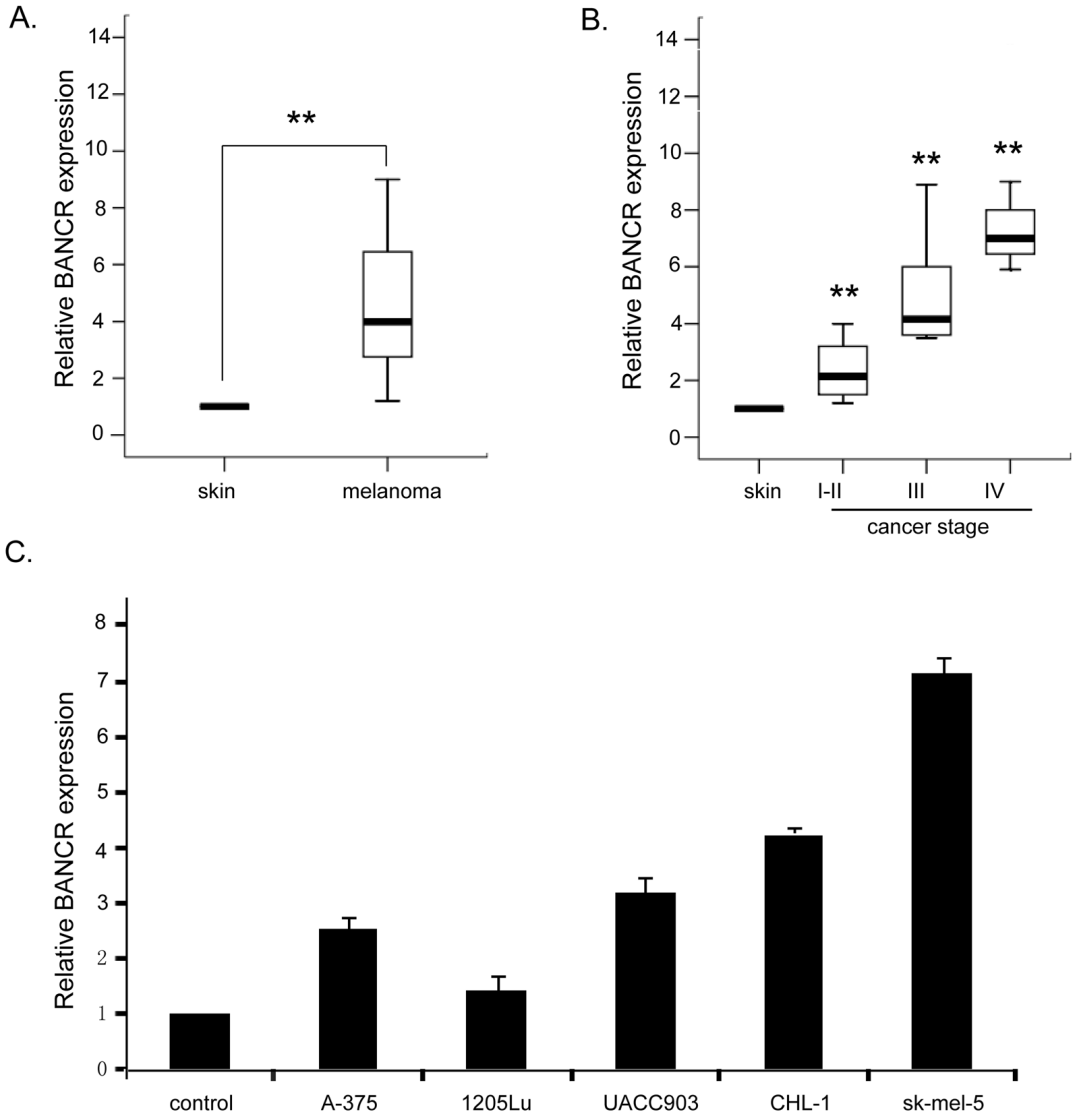
Increased expression of BANCR in both malignant melanoma tissues and cell lines. (A) Increased BANCR expression in malignant melanoma tissues compared to control skin tissues. (B) BANCR expression increased with clinical stages of malignant melanoma. (C) Significant high expression of BANCR in five melanoma cell lines in comparison with control skin tissues pooled from 3 controls with melanocytic nevus. (** P<0.01).

Expression of BANCR in a total of 103 human tissue specimens of malignant melanoma with various cancer stages and melanoma cell lines was detected using SYBR green quantitative PCR analysis. A significantly increased level of BANCR was seen in patients with malignant melanoma (Figure 1A, P = 0.007), compared with the levels detected in age/gender-matched controls with melanocytic nevus. Patients were then grouped by clinical cancer stages. Differences between controls and patients with stages I/II, stage III, and stage IV tumors were also statistically significant (Figure 1B, P<0.001). A direct correlation between clinical cancer stages and BANCR was noted. There was a significantly positive correlation between BANCR expression and tumor stages (P<0.05), while no correlation was observed between BANCR expression and age, gender, localization or tumor size. Then we extended our test to five human melanoma cell lines. The total five cell lines showed a notable overexpression of BANCR compared to the control skin tissue pooled from 3 controls with melanocytic nevus (Figure 1C, P<0.001).

### Knockdown of BANCR inhibited melanoma proliferation *in vitro* and *in vivo*

**Figure 2:**
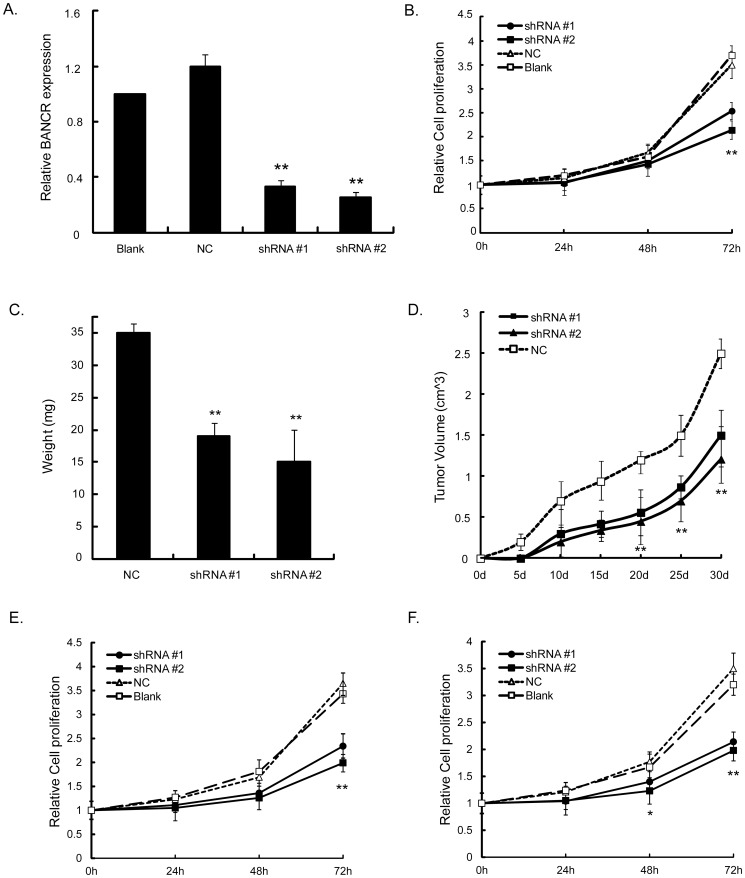
Effects of BANCR on proliferation melanoma cells. (A) Expression of BANCR was significantly silenced by transfecting sk-mel-5 cells with shRNA. Loss of BANCR expression significantly inhibited (B) proliferation of sk-mel-5 cells, tumor growth, including (C) tumor weight and (D) volume in nude mice. Loss of BANCR expression significantly inhibited proliferation of UACC903 (E) and CHL-1 (F) cells, (**P<0.01, Figure is representative of 3 experiments with similar results.)

We selected sk-mel-5 cells to perform further assays to test whether BANCR was functionally involved in malignant melanoma tumorigenesis. BANCR shRNA was stably transfected into the sk-mel-5 cells and expression of BANCR in these clones was confirmed by PCR analysis (Figure 2A). shRNA transfectant cells exhibited significantly decreased proliferation compared with either NC shRNA transfectants cells or parental cells (P<0.01, Figure 2B). To examine whether downregulation of BANCR could inhibit tumorigenicity of sk-mel-5 cells *in vivo*, a xenograft tumor model was applied. As shown in Figure 2C and 2D, the shRNA transfectant cells developed tumors with much smaller volumes (1.27±0.13 for shRNA #1 and 1.01±0.14 for shRNA #2 *vs*. 2.51±0.17 at the fifth week, P<0.01) and lighter weight (15±2.1 for shRNA #1 and 13±3.5 for shRNA #2 *vs*. 35±1.5 at the fifth week, P<0.01) than controls.

Furthermore, we performed the same proliferation assays in UACC903 and CHL-1 cells which also exhibit relatively high expression of BANCR to explore whether the above effect on proliferation seen in sk-mel-5 cells was cell line specific. As shown in Figure 2E and 2F, the inhibitory effects of sh-BANCR transfection on cell proliferation were observed as well. Taken together, it indicated that BANCR regulated cell proliferation, and BANCR depletion impaired proliferation of melanoma cells with high expression of BANCR.

### Knockdown of BANCR inactivated ERK1/2, Raf-1 and JNK, but had no effect on p38 MAPK

**Figure 3:**
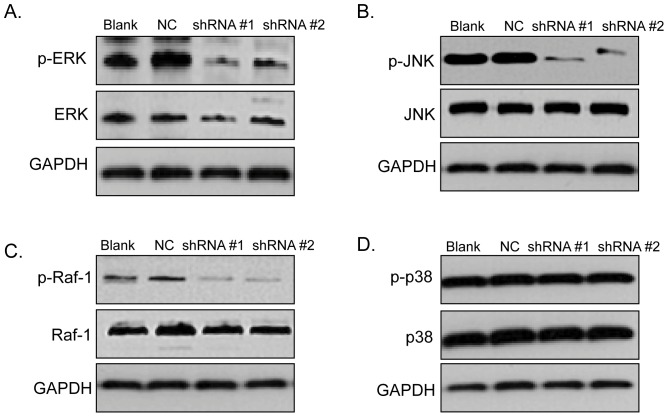
The activities of ERK1/2, Raf-1 and JNK in BANCR silencing sk-mel-5 cells were significantly repressed. Expressions of ERK1/1, Raf-1, p38 and JNK in BANCR silencing sk-mel-5 cells were detected by western blot. Loss of BANCR induced the inactivation of (A) ERK1/2, (B) JNK and (C) the upstream molecule of ERK1/2, Raf-1. (D) However, no activation was observed in p38 MAPK. GAPDH expression was used to normalize for equal loading. Figure is representative of 3 experiments with similar results.

We tested the activation of MAPK pathway to explore the potential underlying mechanisms of BANCR-related low cell growth by western blot. The results showed that ERK1/2 and JNK were inactivated in shRNA transfectant cells compared with NC shRNA transfectant cells (Figure 3A and B). However, no differences of p38 MAPK were observed (Figure 3D). To determine the role of BANCR in the Raf-ERK pathway, we further detected expression of Raf-1, the upstream ERK1/2 activator. And Raf-1 protein levels decreased as well after BANCR was downregulated (Figure 3C).

### BANCR regulated melanoma proliferation synergistically with ERK1/2 and JNK inactivation

**Figure 4:**
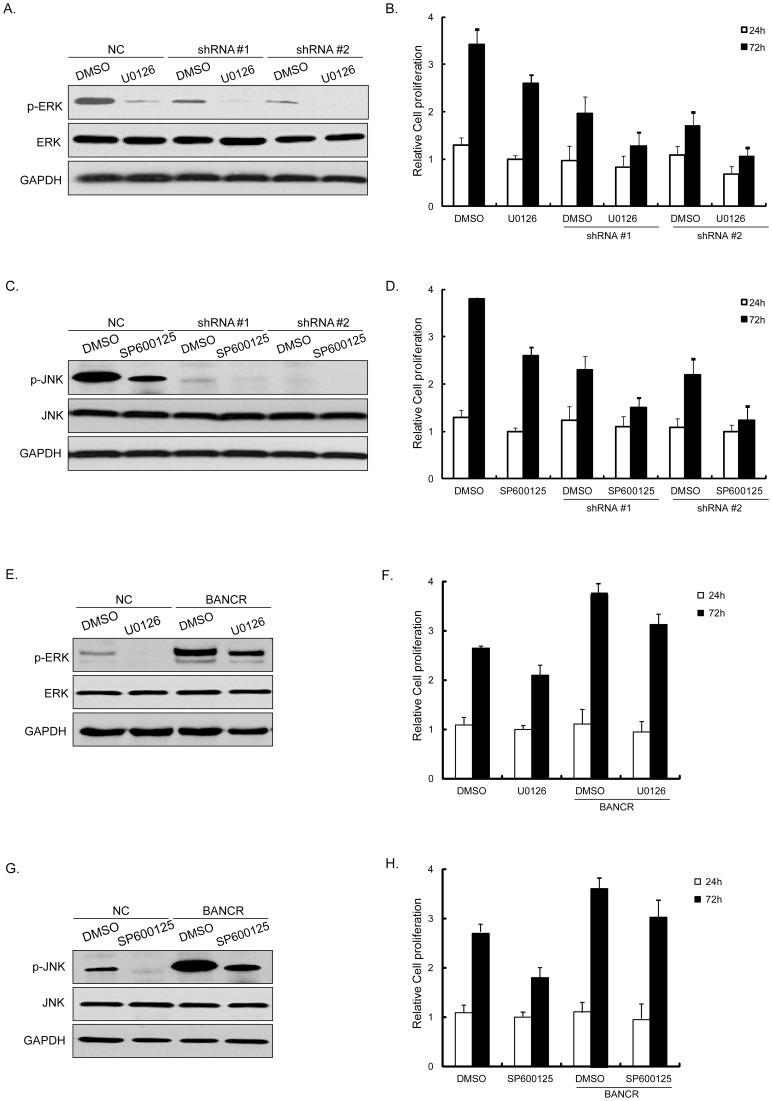
Inactivation of ERK1/2 and JNK participated in BANCR-regulated proliferation. Cells transfected with sh-BANCR or BANCR plasmid were treated with U0126 or SP600125 respectively. Western blot was performed to detect total and phosphorylation changes of ERK1/2 and JNK. U0126 or SP600125 induced significant inactivation of ERK1/2 (A) and JNK (C). Cell proliferation decreased significantly after 72 h (B, D). Note the notable effects induced by combined treatment with shRNA and inhibitors. BANCR activated ERK1/2 (E) and JNK (G) pathways. Inactivation of ERK1/2 and JNK were rescued by overexpression of BANCR. Inhibitory proliferation induced by ERK1/2 and JNK inactivation was ameliorated by BANCR (F, H). Figure is representative of 3 experiments with similar results.

We treated shRNA transfectant cells with specific pharmacological inhibitors U0126 and SP600125 to investigate the effects of ERK1/2 and JNK on BANCR-related proliferation. Western blot was performed to detect total and phosphorylation changes of ERK1/2 and JNK after cells were treated with U0126 and SP600125. As shown in Figure 4, the indicated inhibitors induced significant inactivation of ERK1/2 (Figure 4A) and JNK (Figure 4C). Consistently, cell proliferation decreased significantly (Figure 4B and 4D) after 72 h. To further investigate the interaction of BANCR and ERK1/2 or JNK, we performed rescue experiment by treating BANCR-plasmid transfected cells with U0126 and SP600125. When BANCR was upregulated, ERK1/2 and JNK pathways were both activated. And more importantly, when ERK1/2 and JNK were inactivated by the indicated inhibitors, overexpression of BANCR rescued the inactivation (Figure 4E and 4G). Proliferation assays showed that the inhibitory proliferation induced by ERK1/2 and JNK inactivation could also be ameliorated by BANCR (Figure 4F and 4H).

### Expression of BANCR was associated with the poor prognosis of malignant melanoma patients

**Figure 5:**
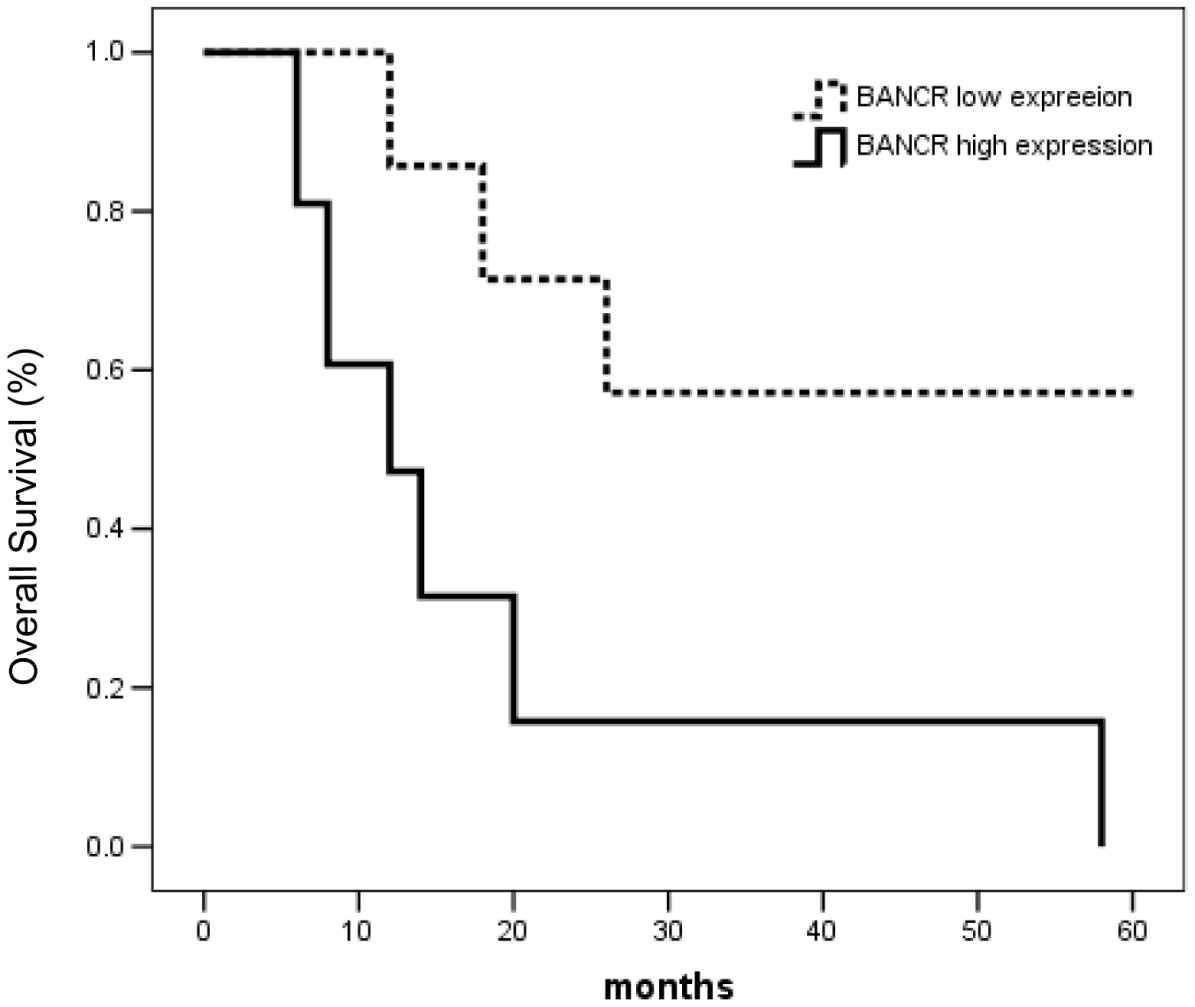
Kaplan-Meier survival curves of malignant melanoma patients relating to the status of BANCR expression.

To investigate the prognostic value of BANCR, the association of BANCR with an overall survival was evaluated using Kaplan-Meier survival curves with the log-rank test. Seventy-two patients were enrolled for this analysis. The follow-up time ranged from 1 to 60 months. The median survival time of the group with higher expression of BANCR was 13.055 months, and the cumulative 1-, 3- and 5-year survival rates were 61%, 40% and 29%, respectively. The median survival time of the lower expression group was 55.021 months, and the 1-, 3- and 5-year survival rates were 86%, 71% and 57%, respectively. The difference between the groups was significant (P<0.01). The univariate survival analysis indicated that the survival rates of patients with higher expression of BANCR was lower than that of patients with lower expression (Figure 5, χ^2^ = 12.826, P = 0.000).

## Discussion

It has been demonstrated by cDNA cloning studies [13] and genomic microarray analysis [14] that more than 90% of the human genome transcriptional products do not code for proteins [15], which are referred to as non-protein coding RNAs (ncRNAs). Among these, lncRNAs, a newly discovered class of noncoding genes, have gained increasing attention because of their crucial roles in gene regulatory processes, such as drug resistance [16], cellular metabolism [17] and apoptosis [18], especially in skin disease and cancer development [19], [20]. Although many new functions have been ascribed to lncRNAs, their functional roles still remain unclear enough. In particular, the involvement of lncRNAs in malignant melanoma tumorigenesis and progression is not fully studied.

In previous study, Flockhart RJ et al. [11] and McCarthy N [12] indicated the potential regulation of BANCR in melanoma cell migration. In the current study, we confirmed the contribution of BANCR in the proliferation of melanoma cells and the potential mechanisms. Our results showed that BANCR was highly expressed in human malignant melanoma cell lines and tissues, and increased with tumor stages. BANCR could repress proliferation of tumor cells both *in vitro* and *in vivo*. Moreover, this inhibitory effect was related with inactivation of MAPK pathway, especially the ERK1/2 and JNK component. Thus BANCR and MAPK pathway can regulate cell proliferation synergistically. Taken together, our finding provided a novel interpretation for the mechanism of BANCR-regulated proliferation in malignant melanoma.

To date, published studies about BANCR are rare and limited. Being overexpressed in melanoma, the oncogenic BRAF-induced BANCR can regulate a set of genes involved in cell migration and is required for full migratory capacity of melanoma cells [20]. However, the underlying mechanism remains obscure. We for the first time demonstrated that BANCR participated in cell proliferation in MAPK pathway-dependent way in detail. The MAPK cascades are key signaling pathways involved in the regulation of cell proliferation, survival and differentiation. Aberrant regulation of MAPK cascades contribute to cancer including malignant melanoma and many other human diseases [21], [22]. The terminal MAPKs are the ERK1/2, JNK, p38 kinases and ERK5. The Raf-ERK1/2 pathway is widely expressed and there has been substantial evidence validating the importance of Raf and ERK in cancer growth and progression [23]. Blocking the pathway by selective inhibitor PD98059 or U0126, inhibits tumor growth in melanoma-bearing mice [24] and induces cell death and abrogates invasive growth of melanoma cells [25]. The JNK pathway is involved in regulating an array of cellular processes, including cell proliferation, migration, and survival, and are thus indispensable for epidermal cancers [26], [27]. Deregulation of the JNK/AP-1 proteins promotes melanoma tumorigenesis [28], and JNK activation is necessary for mitochondrial membrane potential change and apoptosis induced by doxycycline in melanoma cell [29]. Our present results showed that ERK1/2 and JNK were inactivated when BANCR was silenced and vice versa. This inactivation was required for BANCR-regulated proliferation and could be rescued by BANCR upregulation. However, p38 MAPK, which suppresses tumor growth by negatively regulating cell survival and proliferation [30], did not participate in the process.

The RAS/RAF/MAPK pathway is hyperactive in about 30% of human cancers, and activating mutations in key members of this pathway serve as driver mutations in many malignancies [31]. Among which, mutationally activated BRAF has been identified in a variety of cancers recently [32] and it occurs in a non-overlapping occurrence in many cancers such as melanomas, colorectal carcinomas, papillary thyroid carcinomas, serous ovarian carcinomas and lung cancers [21], [33]. Moreover, 90% of melanomas containing the activating mutations in BRAF produce active mutant BRAF^V600E^ protein. BANCR was the mutant BRAF^V600E^-activated long non-coding RNA identified from samples from BRAF-mutant human melanomas. In our study, high expression of BANCR was correlated significantly to shortened survival of patients with malignant melanoma, suggesting that BANCR may be a predictor of poor clinical outcome.

It has been previously shown that ERK1/2 or JNK pathway is a potential target for therapy of cancer [34], [35]. However, the interaction between such pathway inhibitors and cancer is complex. Flores LG 2^nd^ [36] found that therapy with U0126 produced only a transient inhibition of tumor glycolytic activity but did not significantly affect the rate of tumor growth in mice nephroblastoma. Our data showed that BANCR-regulated proliferation was not only ERK1/2-dependent but also JNK-dependent. And the inhibitory effect of BANCR silencing was more remarkable when introduced in combination with ERK1/2 and JNK inactivation induced by pharmacological inhibitors U0126 or SP600125. Moreover, we found BANCR is associated with poor prognosis of patients with malignant melanoma. Taken together, BANCR may be both a new potential target and prognostic factor of malignant melanoma. Further investigation will be required to elucidate this question.
