# Correction: Long Non-Coding RNA BANCR Promotes Proliferation in Malignant Melanoma by Regulating MAPK Pathway Activation

**DOI:** 10.1371/journal.pone.0118728

**Published:** 2015-03-05

**Authors:** The PLOS ONE Staff

The sixth author, Lidao Bao, should be listed as a co-corresponding author with the email address lidao_bao@163.com.
